# The ascent of AKAPs, from architectural elements to kinase anchors: a perspective

**DOI:** 10.1042/BCJ20253085

**Published:** 2025-05-13

**Authors:** Jerome I. Falcone, John D. Scott

**Affiliations:** 1 Department of Pharmacology, University of Washington School of Medicine, 1959 NE Pacific St., Seattle, WA 98195, USA

**Keywords:** evolutionary biology, kinases, protein kinase A, signaling, A kinase anchoring proteins

## Abstract

Protein interaction domains binding to their recognition motifs are the nuts and bolts that hold macromolecular complexes together. Point mutations and gene fusions that drive evolutionary changes in these interactors have created a burgeoning repertoire of protein scaffolds. A-Kinase anchoring proteins (AKAPs) are archetypal signal organizing proteins that compartmentalize protein kinase A (PKA) inside the cell. An amino-terminal docking and dimerization (d/d) domain on the regulatory subunit of the kinase binds with high affinity to an amphipathic helix on the AKAP. This perspective introduces a new group of interactors called docking and dimerization domain interacting proteins that preceded the advent of the AKAP-PKA interface. We also examine various evolutionary paths used by anchoring proteins to gain PKA binding function.

## Introduction

‘Variations on a theme’ are often the realm of composers, such as Mozart, Brahms, Haydn and Benjamin Britten. Yet varying the use of a conserved molecular interface across a range of cellular contexts is also a sound biochemical principle. Modular protein–protein interactions are heavily utilized to connect enzyme binding partners and structural proteins that shape the architecture of the cell [1,2]. Accordingly, interaction modules are a versatile group of protein domains that participate in the assembly of macromolecular machines, cell signaling networks and the subcellular tethering of enzymes. Prototypic examples include Plextrin homology (PH) and Src homology 2 (SH2) domains [3–5]. Both are found in vertebrate, Drosophila, *C. elegans* and yeast proteins. This suggests an ancient origin of fundamental importance to eukaryotic biology. These polypeptide units support protein–lipid and protein–protein interactions to potentiate a variety of cell signaling responses. The PH domain is a highly conserved structure of ∼120 amino acids that binds phospholipids in biological membranes and proteins, including the β–γ subunits of heterotrimeric G proteins [5]. Similarly, SH2 domains are about 100 amino acids that fold to form binding modules that recognize phosphotyrosine on the surface of target proteins [6]. Mutational and gene fusion events underlie the adaptive changes necessary for organisms to develop more elaborate functions and increasingly sophisticated tissue types. Thus, investigating the expansion of modular domains offers a fascinating glimpse into the complex evolution of protein networks.

According to Darwin’s theory of natural selection, mutations that harm the fitness of an organism are selected against. At a molecular level, this leads to functionally important regions of a protein being conserved [7,8]. Conversely, regions with a high degree of variability are sometimes less critical to the protein’s function. These regions can often sustain alterations in amino acids without adversely impacting the organism’s fitness [9]. This process is illustrated in the emergence of ‘docking and dimerization domains’ (d/d domains) [10–12]. These modules were originally discovered within the dimeric regulatory (R) subunits of protein kinase A (PKA). These structures mediate antiparallel dimerization of each peptide chain to create a binding site for A-kinase anchoring proteins (AKAPs) [13–17]. This diverse family of signal organizing proteins functions to sequester PKA with other kinases, phosphatases and the cAMP synthesis machinery at discrete locations in all cells. The importance of AKAP signaling has been demonstrated in several cellular and *in vivo* contexts [18]. These include phosphorylation-dependent modulation of ion channels, regulation of excitatory synaptic transmission and control of steroid hormone biosynthesis [19–21]. More recently, it has been shown that mislocalization of PKA via the loss of AKAP interactions underlies certain endocrine disorders such as adrenal Cushing’s syndrome [22–24]. Hence, second messenger responses that proceed through AKAPs mechanisms represent a means to focus and refine the signaling output of cells [25–27]. The bioinformatic and phylogenetic studies presented in this perspective argue that the origins of the PKA interface with AKAPs can be traced to unicellular organisms, which existed long before the emergence of either the kinase or anchoring proteins [18,25].

## DPY-30 motifs

**Figure 1:**
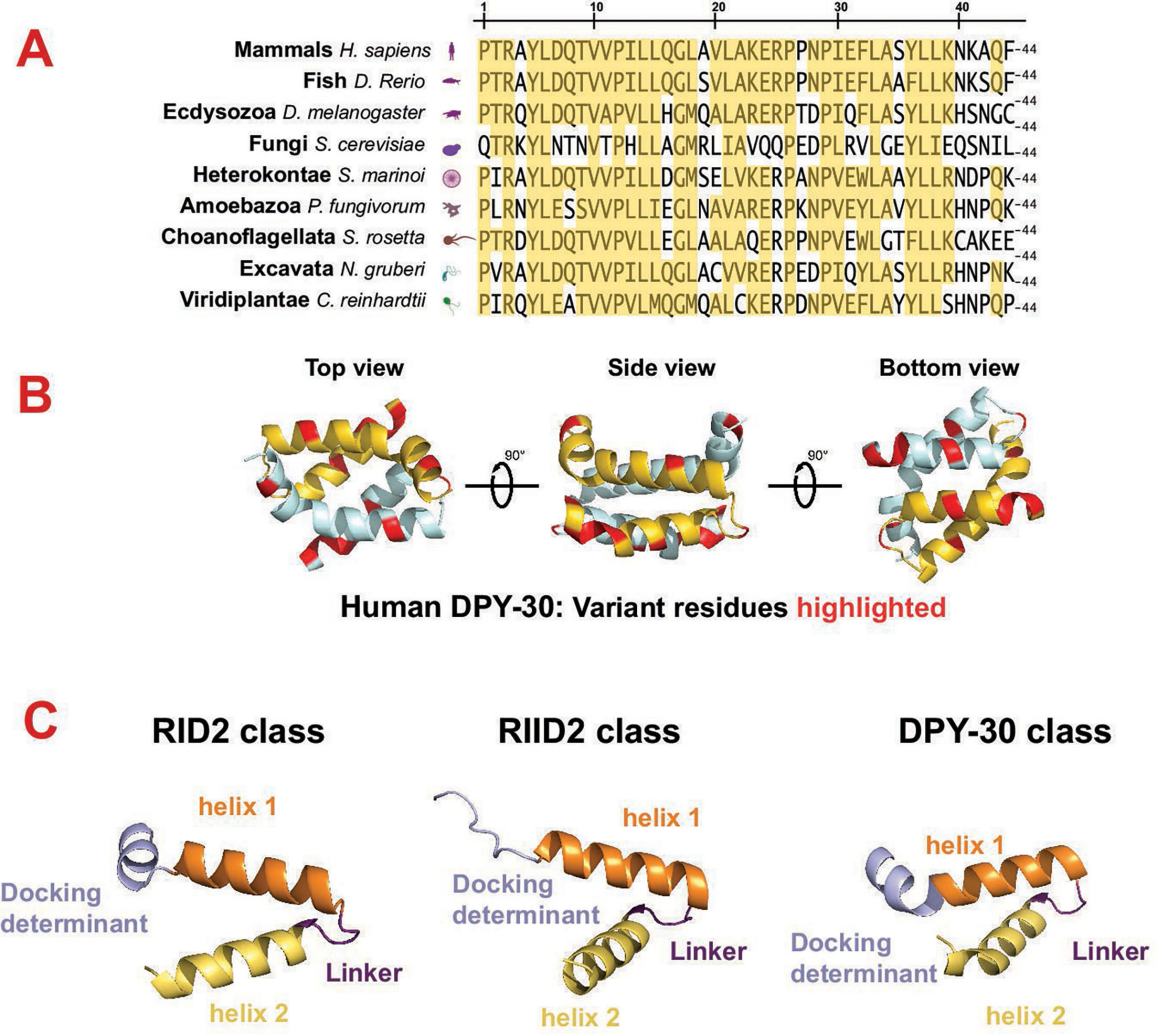
Conservation of the DPY-30 docking and dimerization domain. (**A**) Aligned sequences of DPY-30 d/d domain in mammals, fish, ecdysozoans, fungi, choanoflagellates, heterokonts, amoebozoans, excavates and plants (names and representative members of each philia indicated). Invariant residues are highlighted in yellow. (**B**) Structural models of the DPY-30 d/d domain (perspectives indicated above each stricture). Variant positions highlighted in red. Conserved positions on each protomer are highlighted (yellow and gray). (**C**) Models of three d/d domain class protomers (indicated above each diagram). Docking determinant region (blue), α-helix 1 (orange), hinge region (purple) and α-helix 2 (yellow) are highlighted.

Support for our view comes from analyses of the DPY-30 motif. This docking and dimerization interface is conserved in ∼30 proteins across many phyla (Figure 1A). In C. *elegans,* this d/d domain is an essential element of the dosage compensation machinery. In mammalian cells, DPY-30 is a core component of the COMPASS histone H3K4 methyltransferase complex that regulates aspects of cell proliferation and differentiation [28–30]. Sequence alignment of DPY-30 motifs spanning mammals, insects, choanoflagellates, fungi, amoeba, excavates, heterokonts and plants reveal 40 residue regions of amino acid identity (Figure 1A, yellow). When this information is transposed onto the three-dimensional structure, poorly conserved residues occur in hinge regions and helical bundles that do not contact anchoring helices ([30]; Figure 1B). This is best illustrated when the variant positions (red) are portrayed in top, bottom and side views of the DPY-30 d/d domain (Figure 1B). Conversely, invariant ‘functional’ amino acids occupy the dimer interface and line the docking pocket (Figure 1B). Hence, the DPY-30 motif is a well-preserved d/d domain that exists in species that appeared before the metazoan expansion.

## RID2 and RIID2 proteins

**Figure 2:**
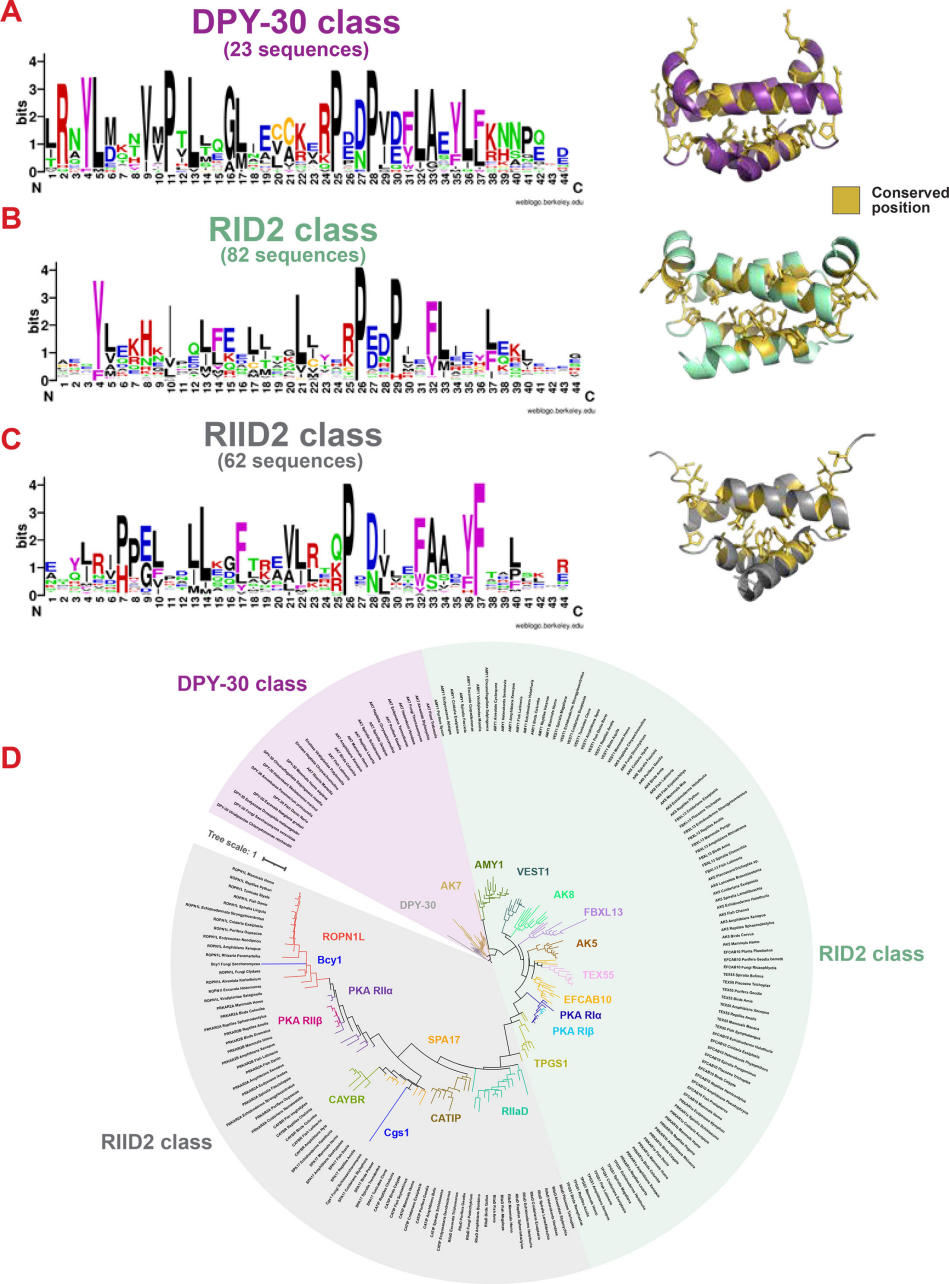
Sequences, conformational topographies and phylogenetic tree of d/d domain protein classes. (**A**) DPY-30-like proteins. (Left) Conservation logo assembled from of 23 orthologs. Amino acids indicated using one letter code. Size of letter indicates prevalence at a given position. (Right) Structural model of DPY-30-like docking and dimerization domain. Conserved positions (gold) are indicated. Variant positions (purple). (**B**) RID2 proteins. (Left) Conservation logo assembled from 82 orthologs. (Right) RIα d/d domain structure. Conserved positions (gold). Variant positions (green). (**C**) RIID2 proteins. (Left) Conservation logo compiled from 62 orthologs. RIIα d/d domain structure. Conserved positions are indicated (gold). Variant positions (gray). Consensus logos in A–C were made from orthologs of d/d proteins in each class acquired by National Center for Biotechnology Information (NCBI) P-BLAST searches. These sequences were curated to ensure that they were not mislabeled, hypothetical, low-quality, or predicted proteins. Sequences were aligned in Mega11 using the Clustalω algorithm and trimmed to only include the d/d domain. The sequence alignments were uploaded to the Weblogo server to generate consensus logos with amino acid colors corresponding to the chemical properties of each residue. (**D**) Phylogenetic tree of 167 d/d containing proteins. Each family of d/d protein arranges into distinct groups on this tree (DPY-30-like – purple, RID2 – green RIID2 – gray). Individual protein families cluster together (colored branches and labels) indicating a divergent pattern of development. The phylogenetic tree was generated from d/d protein orthologs in each class acquired through NCBI P-BLAST searches. These sequences were curated to remove mislabeled, low-quality, hypothetical and predicted proteins, and then aligned in Mega11 with Clustalω. IQtree was used to generate the phylogenetic tree using an ultrafast bootstrap model with 3000 bootstraps. The DPY-30 family was used as an outgroup.

As the utility of d/d domains expanded, variant families emerged (Figure 1C). The DPY-30-like proteins were joined by the RID2 and RIID2 proteins, the latter two being named for folds found in the type I and type II PKA regulatory subunits, respectively [16,31,32]. This collection of 167 sequences each encodes the conserved domain. Although biologically diverse, the anatomy of each protomer is remarkably similar [31]. An N-terminal 8-residue docking determinant (blue) is fused to a 17-residue α-helix (α-helix 1, orange). This is linked via a 4-amino-acid flexible linker (purple) to a 15-residue α-helix (α-helix 2, yellow; Figure 1C). DPY-30-like and RID2 domains incorporate an additional helical extension at their N terminus (Figure 1C, left and mid). In contrast, the corresponding region in the RIID2 proteins is disordered [31] (Figure 1C, right). Sequence logos of the DPY-30 (23 proteins), RID2 (82 proteins) and RIID2 (62 proteins) classes reveal distinguishing features in each domain family (Figure 2A–C). Each class has a proline at a residue that caps α-helix 1 (Figure 2A–C). This imino-peptide bond introduces rigidity to terminate the first α-helix and further orients the second helix in a different plane. This ensures a more constrained interface for docking with binding partners. Each d/d domain family contains a signature amino acid at position 4 (Figure 2A–C). DPY-30 and RID2 proteins contain a tyrosine at this position (Figure 2A and B), whereas branched aliphatic chains (leucine, isoleucine or valine) occupy residue 4 in the RIID2 class (Figure 2C).

Consensus sequence alignments and structural models presented in Figure 2A–C reveal that each d/d domain is tailored toward its unique function. DPY-30-like and RID2 proteins contain two conserved prolines in the linker (capping both the C-terminus of α-helix 1 and the N-terminus of α-helix 2). They also contain a pair of aromatic and branched aliphatic residues in α-helix 2 (Figure 2A and B). These determinants orient and stabilize the core of the docking and dimerization surfaces, respectively. An exclusive property of DPY-30-like proteins is that they have a shorter α-helix 1 than RID2 or RIID2 proteins. This makes the binding surface more compact and reduces the area available for association with amphipathic helices on their binding partners. RIID2 proteins are unique in that the docking region contains aliphatic residues at both positions 4 and 6, the linker region. Also, a single proline caps the end of α-helix 1 (Figure 2C). Other characteristic features include a bent helix for DPY-30-like, a helix with a linker for RID2, and an unstructured region for RIID2 proteins (Figure 2A–C). These symbolic features induce docking to distinct binding partners. In addition, d/d domains fuse with other modular units to generate a repertoire of scaffolding proteins with various functions [31].

We propose that the DPY-30 and the RID2 and RIID2 classes have evolved divergently, a process where proteins originating from a common ancestor develop different traits over time. A phylogenetic tree of d/d domain proteins provides further evidence for their divergent evolution (Figure 2D). The tree was constructed from sequences from representative species in phylogenetically distinct groups, capturing the breadth of variation across orthologs. The tree was rooted using the DPY-30-like family as an outgroup. The three d/d families distribute separately, with RID2 proteins (Figure 2D; green) positioned between the DPY-30-like family (Figure 2D; purple) and the more distant RIID2 family (Figure 2D; gray). A clear pattern of divergence emerges upon examining the relationships between individual proteins (Figure 2D). Clusters of d/d orthologs with short branch lengths maintain separation from other proteins (Figure 2D; colored branches). One exception is the β-forms of PKA regulatory subunits (RIβ and RIIβ) that are nested within the larger groups of the α-forms of the regulatory subunit. Of particular note are the fungal PKA regulatory subunits, Cgs1 and Bcy1 (Figure 2D, blue lines; [33,34]). These cAMP binding proteins are of the RIID2 class, but both contain d/d domain insertions that affect oligomerization. This favors the formation of tetrameric assemblies and precludes binding to anchoring proteins [35]. Both proteins reside in distinct protein clusters but have extremely long branch lengths, inferring considerable evolutionary divergence (Figure 2D**, blue lines).**

### Docking and dimerization domain interacting proteins

All three of the d/d domain classes interact with helical regions that, in some respects, resemble PKA anchoring proteins. This previously unidentified group of interactors does not bind protein kinases. Therefore, we have named this burgeoning family D**/**D domain interacting proteins (DDIPs). This new scaffolding protein class exhibits a broad range of biological roles that include constraining the architecture of motile cilia and flagella, assembly of the nuclear histone methylation machinery and the formation of macromolecular complexes that participate in cell metabolism.

**Figure 3:**
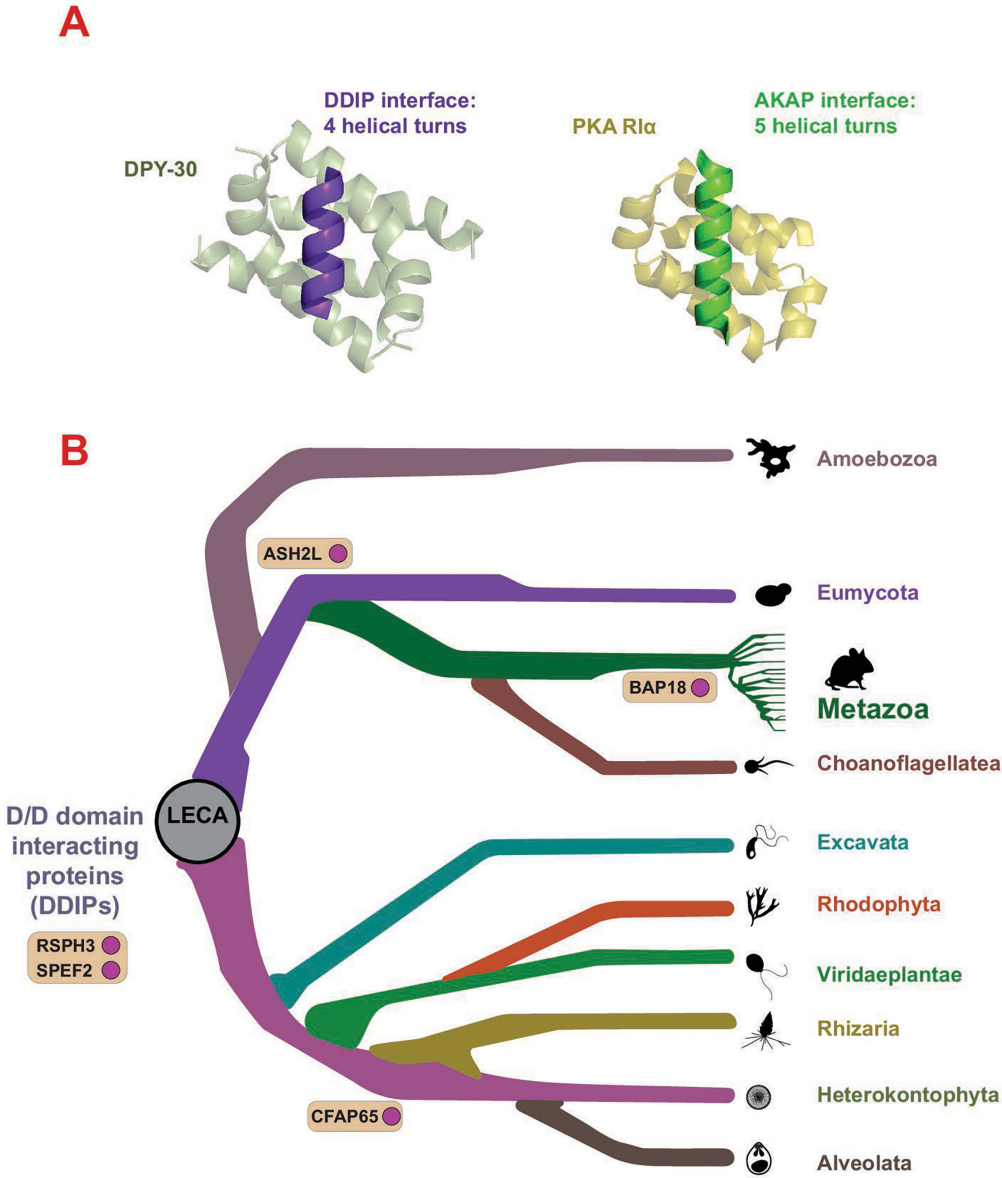
Docking and dimerization domain interacting proteins **(**DDIPs**).** (**A**) Comparative configurations A, (Left). A prototypical DDIP helix (four helical turns, purple) interacting with DPY-30, (Right) the corresponding region of an A-Kinase Anchoring protein (AKAP) (five helical turns, green) binding to RIα. Cladogram charting the emergence of DDIPs across the eukaryotic domain. (**B**) Individual DDIPs (magenta dots) are indicated. Binding partners present at the last eukaryotic common ancestor (LECA) are indicated. Phila where subsequent DDIPs emerged are denoted (magenta dots).

Structural modeling predicts that DDIPs associate with their binding partners in a manner that is distinct from AKAPs. For example, DPY-30 family members only require four turns of an amphipathic helix for high affinity interaction with their binding partners (Figure 3A; purple [29]). In contrast, five helical turns are necessary for R subunits of PKA binding to AKAPs ([13,14,36–38] Figure 3A, green). An evolutionary tree of the DDIP class reveals that most of its members are more ancient than PKA anchoring proteins (Figure 3B). Also, DDIPs are predominantly found in cilia and flagella, where they play a structural role in the axoneme [39]. For example, CFAP65 first appeared in diaphoretickes (a parent clade of plants, diatoms and flagellates) as a structural component of the ciliary axoneme ([39]; Figure 3B). Interestingly, certain proteins originally designated as PKA anchoring proteins, such as AKAP28 could be reclassified as DDIPs, as they bind RIID2 proteins [40]. In keeping with this notion, both CFAP65 and AKAP28 participate in protein–protein interactions that provide rigidity to support dynein motor function in cilia and flagella. These DDIP scaffolding proteins also integrate other signaling elements, such as calcium-responsive components, E3 ligases and adenylate kinases [41]. Interestingly, certain DDIPs are present at the last eukaryotic common ancestor (Figure 3B). ASH2L and BAP18 bind DPY-30, promoting histone methylation and the reading of methylated histones, respectively [42]. Thus, a key finding is that the conserved DDIP interface maintains the integrity of macromolecular machines that coordinate a broader range of biological processes than AKAPs.

### Evolutionary paths of AKAPs

**Figure 4:**
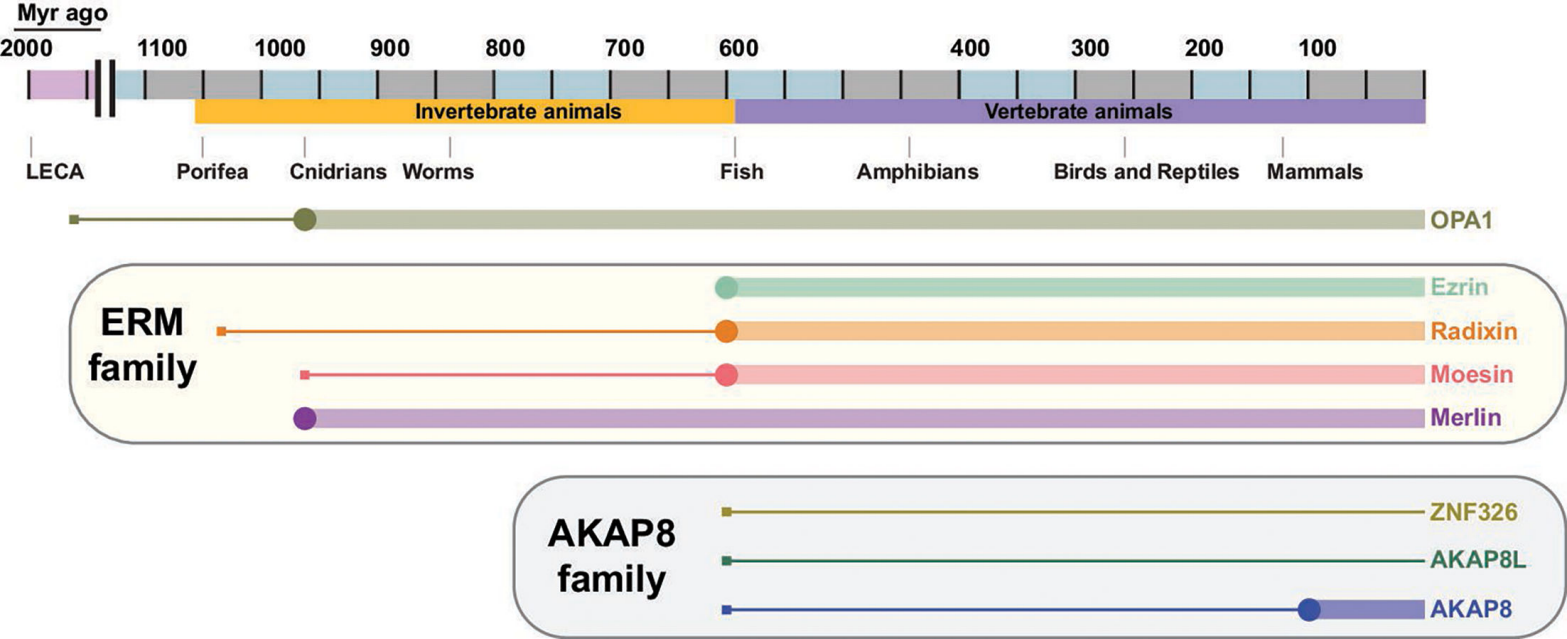
Timeline for the emergence of selected AKAP families. (Top) OPA1 (gray) originated in the opisthokont lineage and gained protein kinase A (PKA) anchoring capability in the metazoan lineage. (Mid) The ERM protein family exhibits varied AKAP development. Merlin (purple) was an AKAP from its origin in the parahoxozoa lineage. Radixin (orange) formed at the last metazoan common ancestor and gained anchoring capabilities at the onset of vertebrates. Moesin (pink) originated in the parahoxozoa lineage and became an AKAP at the start of the vertebrate lineage. Ezrin (mint green) became an AKAP at the vertebrate lineage. (Bottom) AKAP8 (blue) appeared alongside ZNF326 (olive) and AKAP8L (teal) at the start of the vertebrate lineage. AKAP8 became an AKAP in the mammalian lineage, whereas ZNF326 and AKAP8L have no extant AKAP forms.

**Figure 5:**
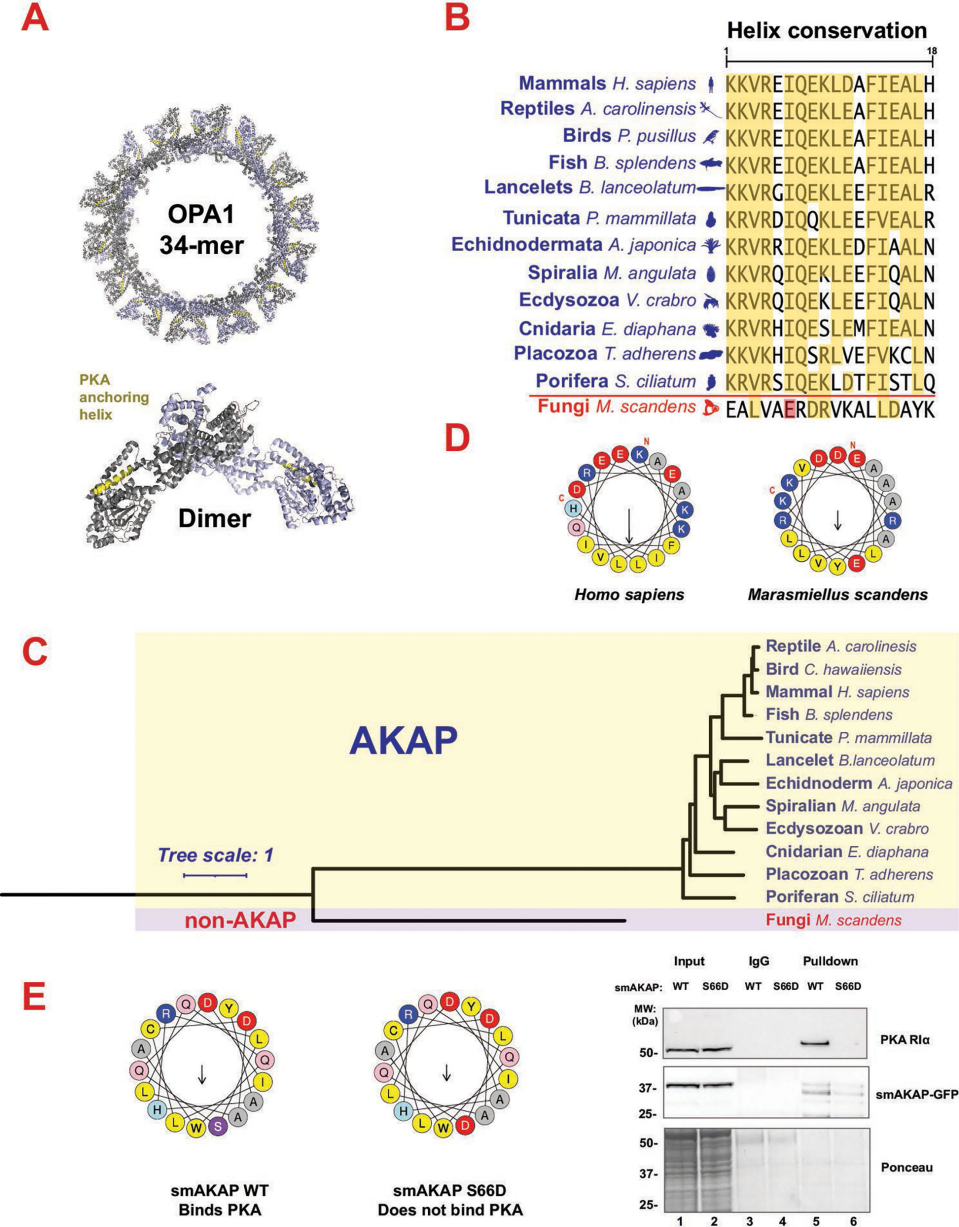
Optic atrophy type 1 protein (OPA1). (**A**) This dynamin-like GTPase sculpts the outer mitochondrial membrane by altering its oligomerization state (Upper) of a 34-mer cyclic assembly to (Lower) antiparallel dimers. The PKA anchoring helix (yellow) is indicated. (**B**) OPA1 became an AKAP at the Metazoan clade. Invariant residues within the PKA anchoring helix (yellow highlighting). Fungal OPA1 sequences are less conserved and contain a glutamic acid (red) that interrupts hydrophobic face of the PKA anchoring helix (**C**). Phylogenetic tree of OPA1 orthologs. AKAP (yellow) metazoan forms cluster together, reflecting the evolutionary relationships of constituent phyla. The phylogenetic tree was generated from OPA1 orthologs in each class acquired through NCBI P-BLAST searches. These sequences were curated to remove mislabeled, low-quality, hypothetical and predicted proteins, and then aligned in Mega11 with Clustalω. IQtree was used to generate the phylogenetic tree using an ultrafast bootstrap model with 3000 bootstraps. Non-AKAP (purple) fungal OPA1 serves as the outgroup. (**D**) Helical wheel projections of human and fungal OPA1 helices show a glutamic acid disrupting the amphipathic helix in the fungal form. (**E** Left) Helicall wheel projections of wild type and mutant smAKAP. (Right) immunoblot of RIa co-precipitation with wildtype smAKAP (lane 5) and smAKAP S66D (Lane 6), where introduction of a charged residue disrupts PKA anchoring. Input (lanes 1 & 2), IgG controls (lanes 3 & 4) and molecular weight markers are indicated.

**Figure 6:**
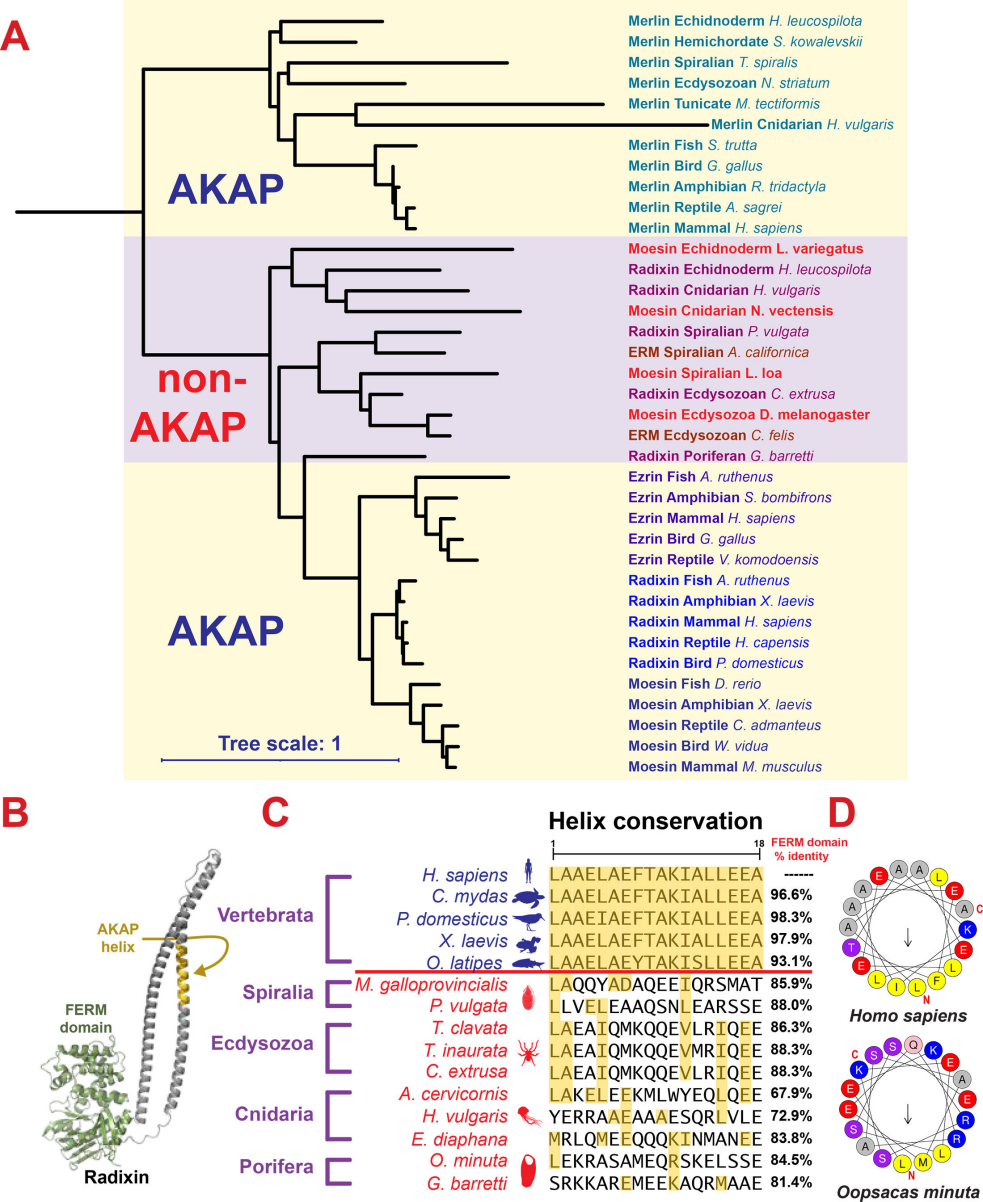
The ERM proteins and Merlin. (**A**) Phylogenetic tree of the ERM family and Merlin depicting asynchronous AKAP development. Merlin orthologs cluster together and are AKAPs (yellow; top). Vertebrate ERM proteins cluster by identity and are functional AKAPs (yellow; bottom). Invertebrate ERM proteins lack AKAP functionality (purple; middle) and cluster by phylogeny rather than protein identity. The phylogenetic tree was generated from ERM protein family orthologs in each class acquired through NCBI P-BLAST searches. These sequences were curated to remove mislabeled, low-quality, hypothetical and predicted proteins, and then aligned in Mega11 with Clustalω. IQtree was used to generate the phylogenetic tree using an ultrafast bootstrap model with 3000 bootstraps. (**B**) Structural model of human Radixin, highlighting its FERM domain (green) and AKAP helix (yellow). (**C**) Alignment of the amphipathic helix region of Radixin orthologs shows an accumulation of neutral mutations in non-AKAP forms, followed by stringent conservation (yellow) once PKA anchoring developed. FERM domain percent identity to human radixin is indicated. (**D**) Helical wheel projections of human (top) and sponge (bottom) demonstrate difference in size of hydrophobic face in AKAP and non-AKAP forms.

**Figure 7:**
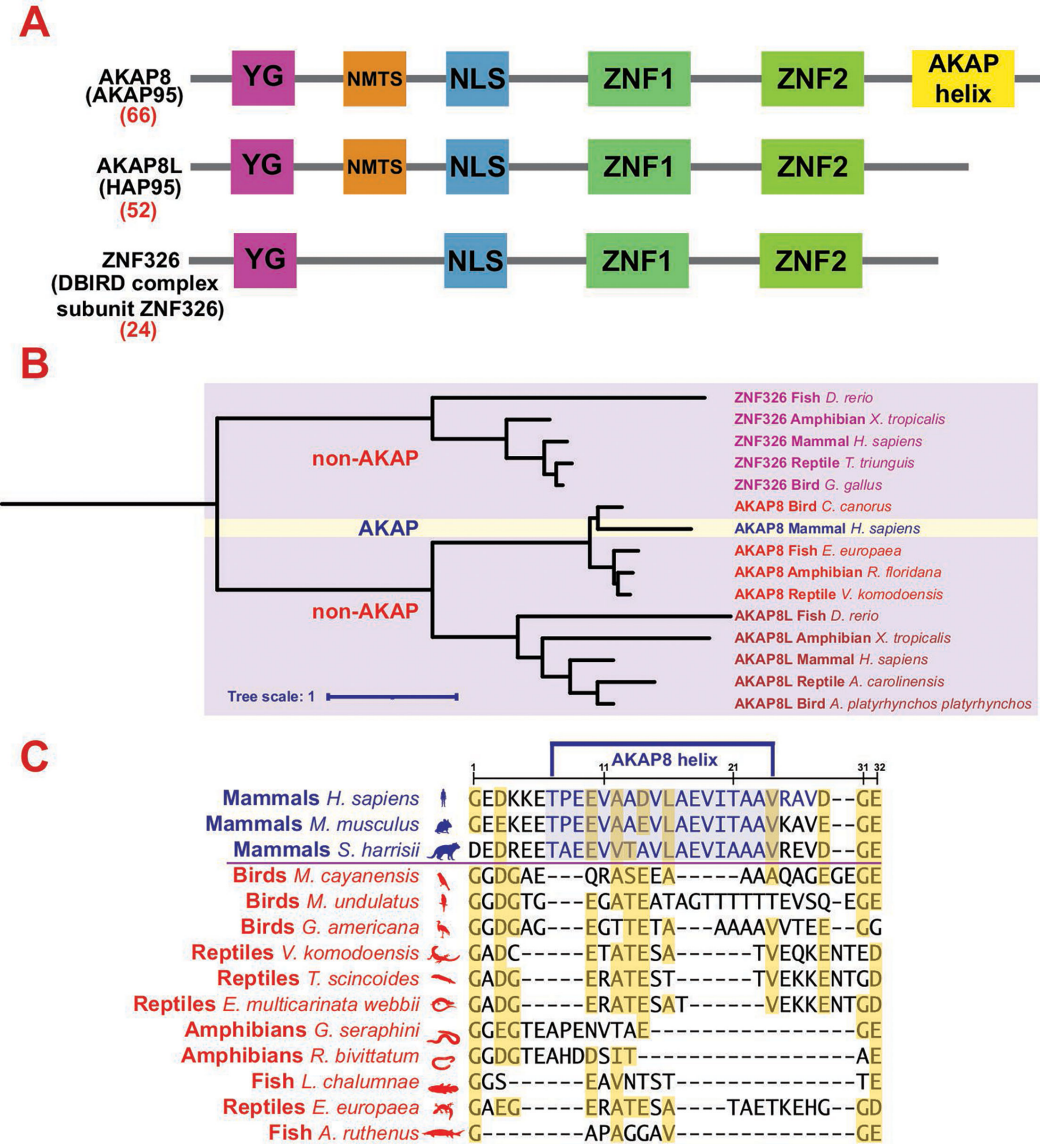
The AKAP95/AKAP8 family. (**A**) Schematic depicting the modular domain organization of AKAP8 (top), AKAP8L (middle) and ZNF326 (bottom) reveals an insertion event of an amphipathic helix converted mammalian that converted AKAP8 into a functional PKA anchoring protein. (**B**) Phylogenetic tree of the AKAP8 family. The three proteins cluster together, but only mammalian AKAP8 forms can anchor PKA (yellow). The phylogenetic tree was generated from AKAP8 family orthologs in each class acquired through NCBI P-BLAST searches. These sequences were curated to remove mislabeled, low-quality, hypothetical and predicted proteins, and then aligned in Mega11 with Clustalω. IQtree was used to generate the phylogenetic tree using an ultrafast bootstrap model with 3000 bootstraps. (**C**) Alignment of AKAP8 orthologs shows that the mammalian helical region (blue) extends into conserved regions in non-mammalian forms (red). The AKAP8 helix arose from an insertion, requiring gaps in the alignments of non-AKAP forms of AKAP8 between conserved flanking regions (yellow).

The sporadic evolution of the PKA binding helix on AKAPs contrasts with the gradual specialization of R subunits from DDIPs. AKAP helices accumulate neutral mutations that ultimately lead to the creation of a PKA binding helix [32,37]. This program of molecular metamorphosis has occurred through a variety of mechanisms. We provide three examples of anchoring proteins that have developed from extant orthologs that do not anchor PKA (Figure 4). The evolutionary path of each protein differs in the time from their origin to the appearance of a functional AKAP form (Figure 4; circle and bold line). First, we introduce how the mitochondrial GTPase OPA1 became a PKA anchoring protein at the onset of the Metazoan clade ([43]; Figure 5). Second, we follow the path of the ezrin, radixin and moesin (ERM) proteins that bring PKA into cortical actin networks that connect to cell membranes ([44–47]; Figure 6). Third, we highlight the AKAP8 family of RNA-binding proteins that exemplify a more recent gain of PKA anchoring function in the form of AKAP95 ([48]; Figure 7). A common feature of each exemplar family is that a gradual accumulation of neutral mutations occurred until a PKA anchoring helix emerged.

## OPA1

Optic atrophy type 1 (OPA1) is a dynamin related GTPase that regulates mitochondrial morphology, cristae structure and oxidative phosphorylation [49]. Mutations in OPA1 underlie optic atrophy, a condition where the optic nerve is damaged and causes blindness [50–52]. OPA1 originated within the opisthokont lineage, as indicated by its presence in both fungal and metazoan forms. It is one of the earliest metazoan AKAPs, coordinating PKA signaling in lipolysis at the outer mitochondrial membrane [43]. OPA1 dimerizes or oligomerizes into a ring-shaped 34-mer that is composed of antiparallel dimeric units ([53,54]; Figure 5A). The amphipathic PKA binding helix (yellow) resides on the exposed outer face of each OPA1 dimer in the ring assembly (Figure 5A, inset).

Analyzing the OPA1 amphipathic helix from fungal to metazoan phyla reveals an invariant region that begins with a conserved pair of basic residues (lysine, lysine/arginine) (Figure 5B). While most AKAPs have hydrophobic residues in this position, OPA1 retains high affinity for both RI (12.5±2.8 nM) and RII (14.0±1.4 nM; [43]). Only fungal OPA1 lacks this region and does not anchor PKA (Figure 5B and C). A phylogenetic tree of OPA1 orthologs further highlights the evolutionary distance between the fungal and metazoan forms (Figure 5C). The fungal OPA1 helix diverges with aspartic acid at position 6 (Figure 5B and D). We and others have shown that introduction of a glutamic acid at this position in the amphipathic helix is sufficient to prevent PKA binding in other AKAPs ([55]; Figure 5E). These latter studies exemplify the impact of inserting a single charged residue in the AKAP helix as a negative determinant for PKA anchoring.

## ERM proteins and merlin

The ERM proteins and their close relative merlin link the plasma membrane to the actin cytoskeleton to control cell shape [56]. Unlike the previous example, only about half of vertebrate ERM proteins have gained AKAP function. This is best demonstrated in a phylogenetic tree of this family (Figure 6A). All merlin orthologs cluster together and anchor PKA, while metazoan ERM proteins form separate groups (Figure 6A). Invertebrate ERM proteins cluster by phylum, with cnidarian, ecdysozoan and spiralian forms, showing closer relationships. Structurally each ERM family member shares a similar domain organization consisting of a globular FERM domain fused to an extended helical stalk ([57]; Figure 6B) .

The AKAP helix emerged within the coiled-coil stalk of ERM proteins (Figure 6B, yellow). By aligning the AKAP helix sequences of radixin across metazoan phyla, we observe a gradual accumulation of neutral mutations leading up to the vertebrate AKAP form (Figure 6C; red). As with OPA1, once the helix acquires PKA-anchoring capability, it becomes highly conserved (Figure 6C; blue). To illustrate this point, each sequence is paired with its percent identity to the *Homo sapiens* FERM domain, highlighting that even distantly related orthologs, such as those from poriferans, maintain a much higher degree of conservation (Figure 6D). Lastly, a helical wheel comparison of the human radixin anchoring helix with a poriferan ortholog from *Oopsacas minuta* reveals a key structural distinction. The human helix features an extended hydrophobic face that enables PKA binding (Figure 6D, top), whereas the poriferan version has fewer hydrophobic residues, thereby rendering it insufficient for PKA anchoring. Hence, the transition of ERM proteins toward becoming A kinase anchoring proteins is partial, whereas merlin has always had the capacity to anchor PKA.

## The AKAP95/AKAP8 family

The AKAP95/AKAP8 family highlights a different pathway for PKA anchoring evolution. This anchoring protein gained an amphipathic helix via insertion. AKAP95, the first member of the family to be identified, was discovered in a protein interaction screen using radiolabeled RIIa [58–60]. Subsequently, other AKAP8 family members were identified, including ZNF326, AKAP8L/ha95 and AKAP95. All of these proteins are involved in RNA splicing [58,60–66]. These proteins share common domains, including a YG domain for RNA binding and phase condensation, a nuclear membrane targeting sequence (AKAP8 and AKAP8L only), a nuclear localization sequence and two zinc fingers [67] (Figure 7A). Only mammalian AKAP95 orthologs possess an amphipathic helix (Figure 7B). Alignment of AKAP95 orthologs suggests that the helix was inserted, as flanking regions are conserved in non-PKA anchoring orthologs (Figure 7C). Moreover, acquisition of a PKA anchoring helix in AKAP95 is a relatively recent evolutionary event as this anchoring protein is only found in mammals (Figure 7). Interestingly, the PKA anchoring role of AKAP8/AKAP95 remains somewhat controversial. This anchoring protein resides in the nucleus and, hence, is partitioned from PKA that is exclusively cytoplasmic during most of the cell cycle [18]. Hence, the PKA anchoring function of AKAP95 may only occur during mitosis at nuclear envelope breakdown [58,60,68,69]. Intriguingly, the PKA-binding domain of AKAP95 is essential for direct interaction with DPY-30 [70]. This anchoring protein may function as both an AKAP and a DDIP.

## Conclusion

**Figure 8:**
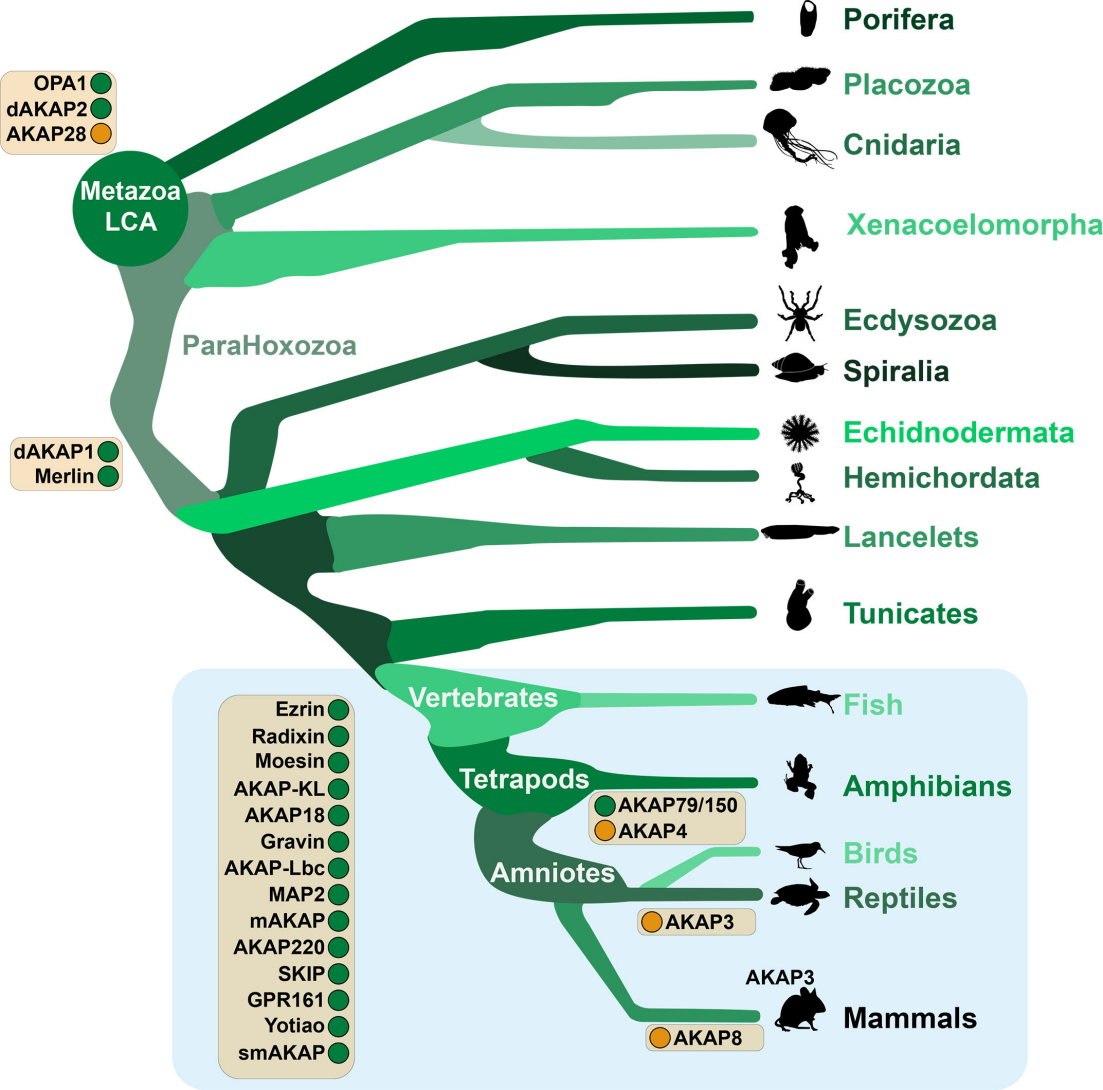
Metazoan cladogram of AKAP origins. Three AKAPs (OPA1, dAKAP2 and AKAP28) were present in the last common metazoan ancestor. Two additional AKAPs (dAKAP1 and Merlin) arose in the parahoxozoa clade. The vertebrate clade experienced an expansion in AKAP diversity, with the 18 anchoring proteins originating in this lineage. AKAPs, which are only known to bind PKA are marked with green dots and AKAPs with reported anchoring of other d/d domain proteins are marked with orange dots.

AKAPs predominantly evolved in metazoans in response to the appearance of anchorable PKA regulatory subunits. This is perhaps best illustrated in the evolutionary tree presented in Figure 8. Three anchoring proteins (OPA1, dAKAP2 and AKAP28) were present at the last common ancestor before the metazoan expansion (Figure 8). Of note is that merlin emerged around the appearance of the ParaHox genes which pattern the anterior–posterior development in most animals [71]. This process requires close coordination with the actin cytoskeleton and is very much in keeping with merlin’s role in maintaining the structural integrity of cells [72]. Also, merlin is an essential gene, whereas its relatives ezrin, radixin, and moesin are non-essential genes [72]. The loss of one ERM protein can be successfully complemented by increased expression of another member of the family [73]. Most AKAPs can only be traced to vertebrates, where an expansion of 16 anchoring proteins were utilized to coordinate spatial integration of cAMP signaling events ([25]; Figure 8; blue-tinted box). This time frame was where certain ERM proteins began to take on PKA anchoring function. In addition, organelle specific anchoring proteins such as D-AKAP1 (mitochondria), AKAP79 (plasma membrane), AKAP220 (vesicles), MAP2 (microtubules), AKAP450 (centrosome/golgi) or AKAP-Lbc (cytoskeleton) began to appear ([20,74–80]; Figure 8).

Another emerging feature is that certain scaffolding proteins may fall under both the DDIP and AKAP designations. For example, AKAP14 and AKAP28 are structural elements in motile cilia that not only bind R subunits but interact with other RIID2 proteins, such as SPA17A and RopN1 [25,31,81,82]. Likewise, AKAP95 retains the ability to bind RII and DPY-30 depending on which subcellular compartment this anchoring protein occupies [40,70]. These anchoring proteins are components of motile cilia, sperm flagellum and radial spoke proteins that control axoneme motility in *Chlamydomonas reinhardtii* [39,83–85]. Most of these AKAPs were discovered by interaction cloning strategies that used the RII-overlay, a far-Western blot procedure as the screen. Consequently, these d/d domain interacting proteins may have been inadvertently misassigned as PKA anchoring proteins [36,86]. This is an understandable consequence as most, if, not all, DDIP proteins utilize the same mode and directionality of interaction with their binding partners as AKAPs. It seems quite likely that new variations of DDIP interfaces that fine tune cellular behavior will make their début soon.

## Abbreviations

AKAP: A-kinase anchoring protein
DDIPs: D/D domain interacting proteins
OPA1: Optic atrophy type 1
PH: Plextrin homology
PKA: protein kinase A
SH2: Src homology 2
